# Comparative efficacy and safety of quetiapine versus other atypical antipsychotics for behavioral and psychological symptoms in Alzheimer’s disease: a systematic review and meta-analysis

**DOI:** 10.3389/fpsyt.2026.1887284

**Published:** 2026-10-26

**Authors:** Jun Wang, Wanyue Chen, Zhihua Zhang

**Affiliations:** 1 Department of Emergency, The Quzhou Affiliated Hospital of Wenzhou Medical University, Quzhou People′s Hospital, Quzhou, Zhejiang, China; 2 Department of Psychiatry, The Third Hospital of Quzhou, Quzhou, Zhejiang, China; 3 Department of Medical Affairs, The Third Hospital of Quzhou, Quzhou, Zhejiang, China

**Keywords:** Alzheimer disease, behavioral and psychological symptoms of dementia, extrapyramidal symptoms, meta-analysis, quetiapine

## Abstract

**Background:**

The management of behavioral and psychological symptoms of dementia (BPSD) in Alzheimer’s disease (AD) remains a significant challenge. While atypical antipsychotics are used, their comparative efficacy and safety, particularly for quetiapine, are not well-established within a homogeneous AD population. This meta-analysis aimed to directly compare the efficacy and safety of quetiapine versus other active pharmacological agents (primarily risperidone and olanzapine) for BPSD specifically in patients with AD.

**Methods:**

A systematic search of PubMed, Embase, Cochrane Library, Web of Science, CNKI, and WanFang databases was conducted up to March 15, 2024, for randomized controlled trials (RCTs). The primary efficacy outcome was treatment failure (lack of clinical improvement). Safety outcomes included specific adverse events. Data were pooled using random-effects models to calculate odds ratios (ORs) with 95% confidence intervals (CIs).

**Results:**

Twenty-five RCTs involving 2143 participants were included. The pooled analysis of 16 studies (n=1384) demonstrated that quetiapine was associated with significantly lower odds of treatment failure compared to other atypical antipsychotics (OR 0.66, 95% CI 0.49 to 0.89, P = 0.006). In pre-specified subgroup analyses, quetiapine showed significant superiority over risperidone (OR 0.50, 95% CI 0.33 to 0.75, P = 0.0009) and a non-significant trend favoring quetiapine over olanzapine (OR 0.90, 95% CI 0.58 to 1.38, P = 0.62). Regarding safety, quetiapine was associated with a substantially and significantly lower risk of extrapyramidal symptoms (EPS) (OR 0.17, 95% CI 0.09 to 0.33, P<0.00001). No significant differences were found for other common adverse events such as somnolence, dizziness, or constipation.

**Conclusion:**

This meta-analysis suggests that quetiapine may offer a potential efficacy advantage for alleviating BPSD in patients with AD, particularly when compared to risperidone, while demonstrating comparable overall effectiveness to other atypicals. Its most distinguishing feature is a substantially lower risk of extrapyramidal symptoms. Interpretation of these findings must be tempered by the moderate risk of bias in constituent trials, the necessary harmonization of disparate efficacy metrics, and the scarcity of long-term safety data. Quetiapine may serve as a neurologically better-tolerated option when non-pharmacological strategies are insufficient, pending careful, time-limited use.

## Introduction

Alzheimer’s disease (AD), the most common cause of dementia, represents a profound global health challenge characterized by progressive cognitive decline and functional impairment. However, the clinical presentation and burden of AD extend far beyond memory loss. A majority of patients, estimated at up to 90% over the course of the illness, develop Behavioral and Psychological Symptoms of Dementia (BPSD), also known as neuropsychiatric symptoms (NPS) (1). This heterogeneous syndrome encompasses agitation, aggression, psychosis (delusions and hallucinations), apathy, depression, anxiety, and aberrant motor activity (2). BPSD is a primary determinant of caregiver distress, accelerated institutionalization, reduced quality of life for patients and families, and increased direct and indirect healthcare costs (3, 4). Effectively managing BPSD is therefore a critical, yet complex, component of comprehensive AD care (5).

The initial management of BPSD strongly emphasizes non-pharmacological interventions, which are recommended as first-line strategies by major clinical guidelines (6, 7). These include person-centered care approaches, caregiver education and support, environmental modifications, and structured activity therapies. While essential and often beneficial, these interventions may have limited efficacy for severe, persistent, or dangerous symptoms, such as significant aggression or psychotic features that pose a risk to the patient or others. In such scenarios, pharmacological treatment becomes a necessary consideration.

The pharmacological landscape for BPSD is marked by significant limitations and caution. While cholinesterase inhibitors and memantine, the standard treatments for AD cognitive symptoms, may have modest benefits for certain BPSD domains, their effect size is often insufficient for acute or severe agitation and psychosis (8). This therapeutic gap has historically led to the widespread off-label use of antipsychotic medications. Both typical (e.g., haloperidol) and atypical (second-generation) antipsychotics have been employed. However, their use in older adults with dementia is fraught with well-documented and serious risks. Meta-analyses and regulatory warnings have consistently associated antipsychotic use in this population with an increased risk of cerebrovascular events, sedation, metabolic disturbances, and a nearly two-fold increase in all-cause mortality (9, 10). Consequently, regulatory agencies like the U.S. Food and Drug Administration (FDA) have issued black-box warnings, and clinical guidelines universally advocate for their judicious, time-limited use at the lowest effective dose, with frequent re-evaluation (6, 11).

This context of high need and significant risk creates a pressing clinical dilemma: when pharmacological intervention for severe BPSD is unavoidable, which agent presents the most favorable balance between efficacy and tolerability? Among atypical antipsychotics, risperidone and olanzapine have the most evidence for efficacy in aggression and psychosis in dementia, but they carry substantial risks, particularly for metabolic side effects (olanzapine) and extrapyramidal symptoms (EPS) or cerebrovascular events (risperidone) (12, 13). Quetiapine, another atypical antipsychotic, is also frequently used in this setting. Its pharmacodynamic profile, with lower affinity for dopamine D2 receptors and higher histamine H1 receptor antagonism, suggests a potentially different side effect profile, possibly with a lower risk of EPS, which is a major concern in the elderly (14).

Despite its common use, the evidence base for quetiapine’s specific role in AD-related BPSD remains less consolidated and more ambiguous compared to risperidone or olanzapine. Existing randomized controlled trials (RCTs) on quetiapine have yielded mixed results regarding its efficacy. Furthermore, many studies are limited by small sample sizes, short durations, heterogeneous patient populations, and the use of different active comparators or placebo. Earlier meta-analysis have attempted to synthesize this evidence, but they often combine different types of dementia or lack direct, head-to-head comparative analyses against other first-line atypical antipsychotics like risperidone and olanzapine specifically in AD populations (15). This evidence fragmentation creates uncertainty for clinicians. Is quetiapine as efficacious as risperidone or olanzapine for managing BPSD in AD? Does its theoretical pharmacological profile translate into a measurably superior safety and tolerability outcome, especially concerning critical endpoints like EPS, in direct comparisons? A comprehensive, up-to-date, and focused synthesis of the evidence is urgently needed to clarify quetiapine’s relative position in the treatment algorithm.

Therefore, we conducted this systematic review and meta-analysis of RCTs with the primary objective of directly evaluating the comparative efficacy and safety of quetiapine versus other active pharmacological agents (primarily other atypical antipsychotics) for the management of BPSD in patients diagnosed specifically with Alzheimer’s disease. By focusing on head-to-head comparisons within a homogeneous AD population, we aim to provide a clearer, more nuanced evidence synthesis. This work seeks to inform clinical decision-making by answering whether quetiapine offers a comparable efficacy benefit with a potentially more benign adverse effect profile, particularly regarding neurologic tolerability, thus helping clinicians navigate the difficult risk-benefit calculus inherent in treating severe BPSD.

## Methods

### Search strategy and data sources

A comprehensive and systematic literature search was performed to identify all relevant randomized controlled trials (RCTs). The following electronic databases were searched from their inception until March 15, 2024: PubMed, Embase, the Cochrane library, Web of Science, China National Knowledge Infrastructure (CNKI), and WanFang Data. The search strategy utilized a combination of Medical Subject Headings (MeSH) terms and free-text keywords related to three core concepts: (1) Alzheimer’s disease, (2) behavioral and psychological symptoms of dementia (BPSD), and (3) quetiapine. Key search terms included: “Alzheimer Disease”, “Dementia”, “Behavioral Symptoms”, “Agitation”, “Psychomotor Agitation”, “Quetiapine”, “Seroquel”, and “Randomized Controlled Trial”. The search strategy was tailored for each database. No language restrictions were applied initially. Additionally, the reference lists of all included studies and relevant review articles were manually screened to identify any potentially eligible publications not captured by the electronic search. This systematic review and meta-analysis was conducted in accordance with the Preferred Reporting Items for Systematic Reviews and Meta-Analyses (PRISMA) 2020 guidelines. Crucially, this research is a purely secondary study. All analyses were based exclusively on aggregate data extracted from previously published, peer-reviewed randomized controlled trials. No primary patient-level data were collected, nor were any institutional or hospital databases accessed for this synthesis.

### Study eligibility and grouping

Selection criteria were defined *a priori* based on established RCT methodologies in Alzheimer’s care. Only randomized controlled trials were eligible. To maintain clinical specificity, included studies were categorized into strict pairwise frameworks: (1) Quetiapine versus Risperidone, (2) Quetiapine versus Olanzapine, and (3) Quetiapine versus placebo. For multi-arm trials, data were extracted pair-wise to prevent participant duplication. This deliberate stratification ensures that comparisons reflect genuine pharmacological differences rather than heterogeneous study designs. Although some eligible trials administered quetiapine or comparators as an adjunct to stable cholinesterase inhibitor therapy, we performed a pre-specified sensitivity analysis excluding combination-therapy studies to verify that the primary efficacy signal was not driven by concomitant cognitive enhancers.

### Eligibility criteria

Studies were selected based on the following PICOS criteria:

#### Population

We included randomized trials that exclusively or predominantly enrolled participants with a confirmed clinical diagnosis of Alzheimer’s disease (per NINCDS-ADRDA, DSM, or ICD criteria) exhibiting behavioral and psychological symptoms of dementia (BPSD). We did not impose restrictions on age, gender, or disease stage in our selection of these published reports.

#### Intervention

We selected trials where oral quetiapine was administered as monotherapy or as an adjunct to a stable cholinesterase inhibitor regimen.

#### Comparator

Trials were required to report on comparators such as other atypical antipsychotics (e.g., risperidone, olanzapine), typical antipsychotics, or placebo.

#### Outcomes

The primary efficacy outcome was treatment response, defined as a reduction of 30% or more from baseline on the Behavioral Pathology in Alzheimer’s Disease Rating Scale (BEHAVE-AD) total score. Although the primary efficacy outcome across most included studies was defined as a ≥30% reduction from baseline on the BEHAVE-AD or Neuropsychiatric Inventory (NPI), the pivotal CATIE-AD trial utilized the Clinical Global Impression of Change (CGIC) scale as its primary outcome measure. To maintain consistency in the pooled analysis, we harmonized the CATIE-AD data by defining treatment failure as a CGIC score of ≥4 (indicating no change or worsening), aligning with the operational definition of ‘lack of clinical improvement’ used in our primary outcome. This approach is consistent with prior meta-analyses integrating CGIC-based endpoints and ensures methodological rigor in combining heterogeneous efficacy metrics. Secondary outcomes included the incidence of specific adverse events: somnolence, dizziness, constipation, extrapyramidal symptoms (EPS), nausea, and tremor.

#### Study design

Only parallel-group or crossover randomized controlled trials (RCTs) were considered for inclusion. Case reports, case series, observational studies, and review articles were excluded.

### Study selection and data extraction

The study selection process was performed independently by two reviewers (WJ and CWY). Initially, all identified records were imported into reference management software (EndNote X9), and duplicates were removed. Subsequently, titles and abstracts were screened against the eligibility criteria. Full-text articles of potentially relevant studies were then retrieved and assessed in detail. Any discrepancies at any stage of the selection process were resolved through discussion or, if necessary, by consulting a third reviewer (ZZH). A standardized, pre-piloted data extraction form was used. For each included publication, we systematically extracted structured information using a standardized form. Extracted items included: first author, publication year, country of origin, trial design, number of randomized groups, and aggregate participant characteristics (e.g., mean age, gender proportions). Rather than collecting raw clinical datasets, we extracted pre-calculated summary statistics, including the number of events for dichotomous outcomes and mean change scores with standard deviations for continuous outcomes, directly from the main texts, tables, and appendices of the published articles. Two reviewers performed data charting independently, followed by cross-verification against the original published sources to eliminate transcription errors.

### Data synthesis

To ensure a transparent and reproducible transition from qualitative literature review to quantitative synthesis, we adopted a strict, pre-specified workflow utilizing exclusively secondary data. First, two reviewers (WJ and CWY) independently screened citations and assessed full texts of published reports against explicit PICOS eligibility criteria, noting that no unpublished datasets were solicited. Second, we performed rigorous, structured aggregate data extraction. Continuous efficacy metrics reported across trials (e.g., NPI scores) were harmonized into uniform, pre-specified responder definitions (≥30% reduction from baseline) to permit valid meta-analytical pooling. Only after this methodological harmonization of published data did we apply random-effects models, ensuring that pooled estimates reflect synthesized evidence rather than primary investigator bias.

### Risk of bias assessment

The methodological quality and risk of bias of the included RCTs were independently assessed by two reviewers (WJ and CWY) using the revised Cochrane Risk of Bias tool for randomized trials. This tool evaluates bias across five domains: (1) bias arising from the randomization process, (2) bias due to deviations from intended interventions, (3) bias due to missing outcome data, (4) bias in measurement of the outcome, and (5) bias in selection of the reported result. Each domain was judged as “low risk”, “some concerns”, or “high risk”. Disagreements were resolved by consensus or arbitration by a third reviewer (ZZH). The overall risk of bias for each study was determined based on the judgments across all domains.

### Statistical analysis

All statistical analyses were performed using Review Manager (RevMan) software, version 5.4, provided by the Cochrane Collaboration. For the primary efficacy outcome, a binary variable was created based on the pre-defined response criterion (≥30% reduction in BEHAVE-AD score). For the primary efficacy analysis, the outcome of ‘treatment failure’ was defined as the absence of a treatment response (i.e., failure to achieve a ≥30% reduction from baseline on the BEHAVE-AD). For the primary efficacy analysis, a binary outcome of ‘treatment failure’ was constructed based on the pre-defined response criteria reported in the source trials. Specifically, the number of individuals experiencing treatment failure in each trial arm was derived by subtracting the reported number of treatment responders from the total number of randomized participants disclosed in the original publication. This allowed us to calculate odds ratios (ORs) comparing the failure rates between quetiapine and control arms using exclusively published aggregate counts, without accessing individual participant data (IPD). Treatment response data were pooled to calculate odds ratios (ORs) with corresponding 95% confidence intervals (CIs). For adverse events, which are also binary outcomes, the pooled risk was similarly expressed as ORs with 95% CIs. The choice of OR was based on its statistical properties in meta-analysis and its common use for such outcomes. Heterogeneity among studies was assessed using the I² statistic and the Cochran’s Q chi-squared test. An I² value greater than 50% or a P-value for the Q test less than 0.10 was considered indicative of substantial statistical heterogeneity. Given the anticipated clinical diversity, a random-effects model was pre-specified for the primary efficacy outcome. For secondary safety outcomes, due to the absence of statistical heterogeneity (I² = 0%), fixed-effect models (Mantel-Haenszel method) were additionally employed to pool odds ratios, ensuring methodological alignment with the visual presentation of forest plots. Pre-specified subgroup analyses were conducted to explore potential sources of heterogeneity and to compare the efficacy of quetiapine versus specific active comparators (risperidone and olanzapine). Publication bias was assessed visually by inspecting funnel plots for asymmetry. A two-sided P-value of less than 0.05 was considered statistically significant for all hypothesis testing, except for tests of heterogeneity where a threshold of 0.10 was used.

## Results

### Study selection

The systematic literature search and study selection process, conducted in accordance with the PRISMA 2020 guidelines, is detailed in **Figure 1**. A total of 454 records were identified through database searching: PubMed (n=63), Embase (n=104), the Cochrane Library (n=2), CNKI (n=148), and WanFang Data (n=137). Before the screening phase, 150 records were removed: 116 were duplicates and 34 were marked as ineligible by automation tools. Subsequently, the titles and abstracts of 304 records were screened. Of these, 188 records were excluded. We sought to retrieve the full-text reports of the remaining 116 articles. However, 34 reports could not be retrieved. Therefore, 82 full-text articles were assessed for eligibility. Among these, 58 articles were excluded for the following reasons: ineligible study design (n=15), intervention not matching the predefined PICO criteria (n=11), outcomes of interest not reported (n=10), data not extractable (n=8), and full text not available in English or Chinese (n=13). Finally, 25 studies met all inclusion criteria and were incorporated into the quantitative synthesis (meta-analysis).

**Figure 1:**
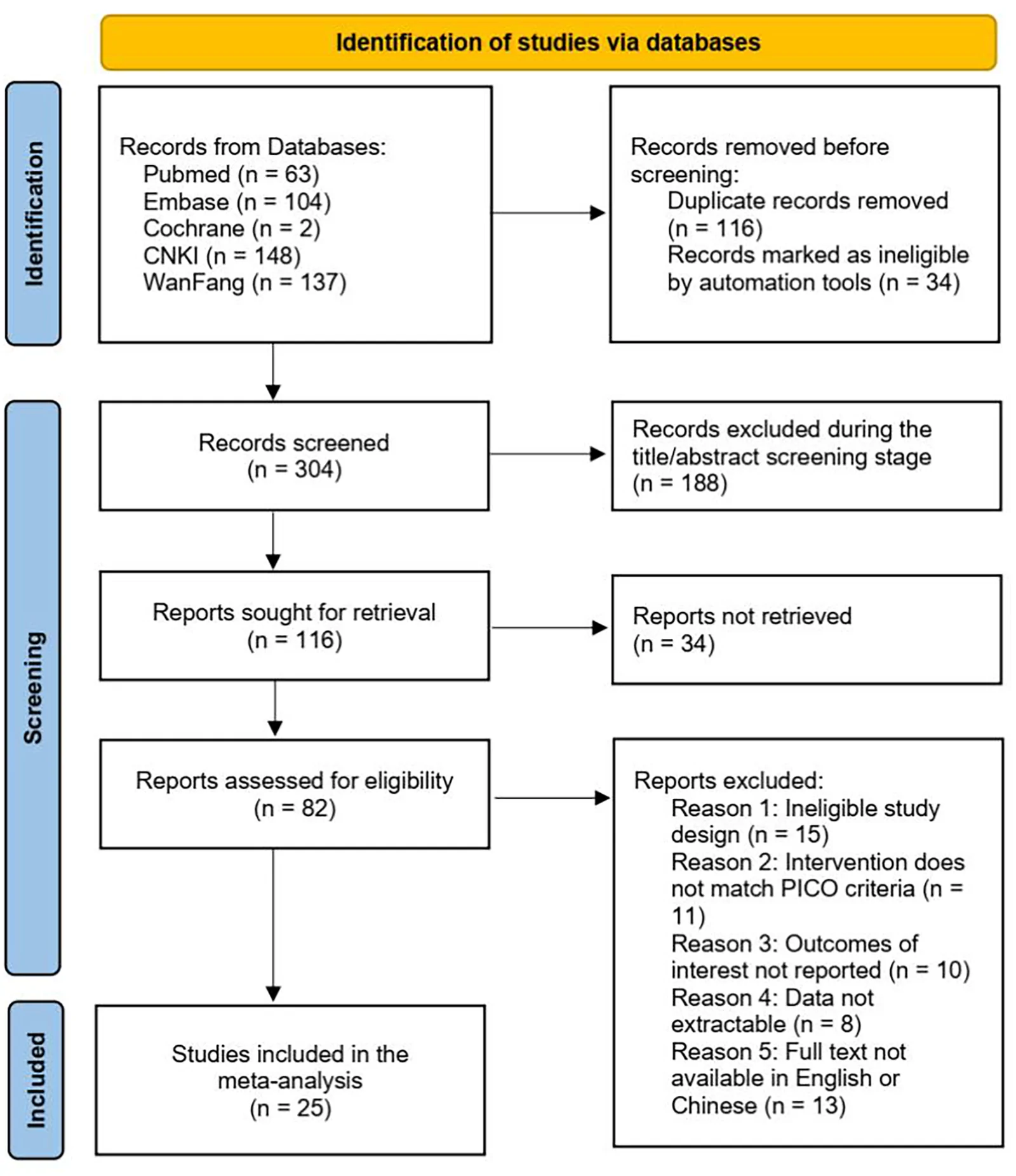
PRISMA flow diagram for the systematic review and meta-analysis.

### Characteristics of included studies

The 25 randomized controlled trials (RCTs) included in the meta-analysis are summarized in **Table 1** (16–40). The publication years spanned from 2005 to 2022. Geographically, studies were conducted in China, United Kingdom, USA, Switzerland, and Israel. The total sample size comprised 2143 participants, with individual study sizes ranging from 22 to 194. Participants were elderly patients diagnosed with Alzheimer’s disease (AD) and associated behavioral and psychological symptoms (BPSD), with mean ages predominantly in the 70-to-80-year range. Diagnosis was based on various criteria, including NINCDS-ADRDA, DSM-IV, ICD-10, CCMD-3, and KCD-10. The intervention group received quetiapine, either as monotherapy or adjunctive to a cholinesterase inhibitor (primarily donepezil or galantamine). Active control groups received other atypical antipsychotics (olanzapine or risperidone), the typical antipsychotic haloperidol, rivastigmine, or placebo. The treatment duration varied from 4 to 36 weeks. The primary efficacy endpoint in most studies was the change in behavioral scores, measured by the Behavioral Pathology in Alzheimer’s Disease Rating Scale (BEHAVE-AD), Neuropsychiatric Inventory (NPI), or Cohen-Mansfield Agitation Inventory (CMAI). Safety outcomes, primarily the incidence of adverse events, were reported in the included studies.

**Table 1:** Characteristics of included studies.

Study (first author, year)	Country	Design	Sample size (Q/C)	Baseline: age, years (mean ± SD); female, n (%)	Diagnostic criteria	Intervention (experimental)	Intervention (control)	Duration (weeks)	Primary outcome (efficacy)	Adverse events reported
Ballard C, 2005 (39)	UK	RCT	32 / 32	Q: 84.3 ± 6.0; 24 (75.0) R: 84.4 ± 7.5; 26 (81.3)	NINCDS-ADRDA	Quetiapine	Rivastigmine (ChEI)	12	NPI, CGIC	Yes (CGIC, specific AEs)
Savaskan E, 2006 (38)	Switzerland	RCT, open-label	11 / 11	Q: 81.6 ± 4.6; 8 (72.7) H: 81.9 ± 6.2; 9 (81.8)	NINCDS-ADRDA	Quetiapine + Galantamine	Haloperidol + Galantamine	5	NPI, Cognitive tests	Yes (Reported)
Schneider, 2006 (40)	USA	RCT	94 / 100	Q:77.3 ± 8.7 yr 50 (53%) O: 78.8 ± 7.3 yr 55 (55%)	DSM-IV NINCDS-ADRDA Probable AD	Quetiapine	Olanzapine	36	Response rate (CGIC ≥ Minimal Improvement) at 12 weeks	Yes (Reported)
Xia JM, 2011 (35)	China	RCT	35 / 35	Q: 76.6 ± 8.1; 20 (57.1) O: 77.8 ± 8.3; 18 (51.4)	ICD-10	Quetiapine	Olanzapine	12	BEHAVE-AD, MMSE	Yes (TESS)
Paleacu D, 2008 (37)	Israel	RCT	20 / 20	Q: 82.9 ± 6.3; 15 (75.0) PBO: 82.6 ± 7.9; 13 (65.0)	DSM-IV	Quetiapine	Placebo	10	CMAI	Yes (Reported)
Zhang YZ, 2020 (18)	China	RCT	31 / 30	Q: 77.40 ± 9.59; 28 (90.3) O: 77.30 ± 10.03; 24 (80.0)	ICD-10	Quetiapine + Donepezil	Olanzapine + Donepezil	6	BEHAVE-AD, MMSE, Hcy	Yes (Reported)
Wu Y, 2010 (36)	China	RCT	40 / 40	Q: 67.2 ± 11.4; 15 (37.5) R: 66.7 ± 9.8; 17 (42.5)	CCMD-3	Quetiapine	Risperidone	6	BEHAVE-AD, MMSE	Yes (TESS)
Yang CY, 2019 (21)	China	RCT, single-blind	26 / 26	Q: 69.21 ± 5.28; 8 (30.8) O: 69.72 ± 5.61; 7 (26.9)	ICD-10	Quetiapine + Donepezil	Olanzapine + Donepezil	8	BEHAVE-AD, ADL	Yes (Reported)
Wang BY, 2020 (19)	China	RCT	65 / 65	Q: 72.8 ± 1.9; 26 (40.0) R: 70.3 ± 2.6; 23 (35.4)	CCMD-3	Quetiapine	Risperidone	8	BEHAVE-AD, Response rate	Yes (TESS)
Li F, 2017 (27)	China	RCT	37 / 37	Q: 68.4 ± 5.5; 17 (45.9) O: 67.9 ± 5.7; 16 (43.2)	ICD-10	Quetiapine	Olanzapine	6	BEHAVE-AD (factors), Response rate	Yes (Reported)
Xu YY, 2019 (22)	China	RCT	40 / 40	Q: 69.3 ± 1.7; 19 (47.5) O: 69.4 ± 1.2; 18 (45.0)	DSM-IV	Quetiapine + Donepezil	Olanzapine + Donepezil	8	BEHAVE-AD, MMSE, ADL	Yes (Reported)
Sun Y, 2012 (34)	China	RCT	37 / 35	Q: 73.68 ± 6.41; 17 (45.9) R: 74.82 ± 5.46; 16 (45.7)	CCMD-3	Quetiapine	Risperidone	8	CMAI, BEHAVE-AD, Response rate	Yes (TESS)
Cheng FF, 2022 (17)	China	RCT	50 / 50	Q: 73.30 ± 10.45; 25 (50.0) R: 73.42 ± 10.97; 26 (52.0)	CCMD-3	Quetiapine	Risperidone	6	BEHAVE-AD, Response rate	Yes (Reported)
Tu SH, 2018 (26)	China	RCT	34 / 48	Q: 74.24 ± 9.27; 20 (58.8) O: 73.88 ± 8.12; 25 (52.1)	NINCDS-ADRDA	Quetiapine	Olanzapine	4	BPRS, MMSE	Yes (QTc, PR interval)
Xu XY, 2013 (32)	China	RCT	44 / 42	Median: 72.8 (overall); NR	CCMD-3	Quetiapine	Risperidone	8	BEHAVE-AD, Response rate	Yes (Reported)
Chen D, 2019 (25)	China	RCT	50 / 50	Q: 73.21 ± 4.22; 22 (44.0) O: 73.09 ± 4.61; 23 (46.0)	CCMD-3	Quetiapine + Donepezil	Olanzapine + Donepezil	12	BEHAVE-AD, MMSE	Yes (Reported)
Wang PJ, 2019 (23)	China	RCT	20 / 20	Q: 60.1 ± 3.2; 9 (45.0) O: 26.8 ± 6.1; 10 (50.0)	ICD-10	Quetiapine + Donepezil	Olanzapine + Donepezil	4	BEHAVE-AD, Response rate	Yes (Reported)
Hu LH, 2017 (28)	China	RCT	60 / 60	Q: 67.1 ± 6.2; 28 (46.7) R: 68.5 ± 6.1; 33 (55.0)	CCMD-3	Quetiapine	Risperidone	8	BEHAVE-AD, BPRS, Response rate	Yes (TESS)
Liao CP, 2013 (33)	China	RCT	35 / 35	Age NR; Gender NR	CCMD-3	Quetiapine	Risperidone	6	BEHAVE-AD, MMSE	Yes (TESS)
Jia X, 2019 (24)	China	RCT	47 / 47	Q: 69.95 ± 2.29; 22 (46.8) O: 71.21 ± 1.71; 24 (51.1)	ICD-10	Quetiapine + Donepezil	Olanzapine + Donepezil	8	BEHAVE-AD, Response rate	Yes (Reported)
Hui ZX, 2015 (31)	China	RCT	32 / 28	Q: 59.73 ± 6.80; 14 (43.8) R: 59.64 ± 6.82; 12 (42.9)	Not specified	Quetiapine	Risperidone	12	BEHAVE-AD (factors), Response rate	NR
Zhang R, 2015 (29)	China	RCT	66 / 62	Q: 68.4 ± 6.3; 28 (42.4) R: 68.6 ± 6.2; 26 (41.9)	CCMD-3	Quetiapine	Risperidone	8	BEHAVE-AD, BPRS, Response rate	Yes (TESS)
Yue J, 2022 (16)	China	RCT*	90 / 90	Q: 66.39 ± 2.36; 40 (44.4) O: 65.07 ± 2.68; 42 (46.7)	CCMD-3	Quetiapine	Olanzapine	12	Response rate	Yes (Reported)
Xu JP, 2015 (30)	China	RCT	40 / 40	Q: 72.13 ± 11.45; 19 (47.5) R: 74.02 ± 10.97; 17 (42.5)	CCMD-3	Quetiapine	Risperidone	6	BEHAVE-AD, Response rate	Yes (Reported)
Zheng XJ, 2019 (20)	China	RCT	32 / 32	Q: 63.26 ± 3.05; 15 (46.9) R: 63.41 ± 3.10; 13 (40.6)	CCMD-3	Quetiapine	Risperidone	6	BEHAVE-AD	NR

AE, Adverse Event; BPRS, Brief Psychiatric Rating Scale; CCMD-3, Chinese Classification of Mental Disorders, Third Edition; CGIC, Clinical Global Impression of Change; ChEI, Cholinesterase Inhibitor; CMAI, Cohen-Mansfield Agitation Inventory; DSM-IV, Diagnostic and Statistical Manual of Mental Disorders, Fourth Edition; H, Haloperidol group; Hcy, Homocysteine; ICD-10, International Classification of Diseases, Tenth Revision; NINCDS-ADRDA, National Institute of Neurological and Communicative Disorders and Stroke–Alzheimer’s Disease and Related Disorders Association; NPI, Neuropsychiatric Inventory; NR, Not Reported; O, Olanzapine group; PBO, Placebo group; Q, Quetiapine group; R, Risperidone group; RCT, Randomized Controlled Trial; TESS, Treatment Emergent Symptom Scale. All values presented in this table represent aggregate data reported in the respective original publications, not primary measurements collected by the authors of this meta-analysis.

### Risk of bias assessment

The methodological quality and risk of bias of the included RCTs were evaluated using the Cochrane Risk of Bias tool, with the results presented in **Figures 2**, **3**. Overall, the risk of bias was judged as moderate. The domains of random sequence generation and allocation concealment were frequently rated as ‘unclear risk’ due to inadequate reporting of methodology in the original publications. The performance bias domain (blinding of participants and personnel) was judged as ‘high risk’ in the majority of studies, as most were open-label or single-blind trials, reflecting pragmatic design considerations in psychopharmacology. In contrast, the domains of detection bias (blinding of outcome assessment), attrition bias (incomplete outcome data), and reporting bias (selective reporting) were generally assessed as ‘low risk’. The risk for other potential biases was often unclear.

**Figure 2:**
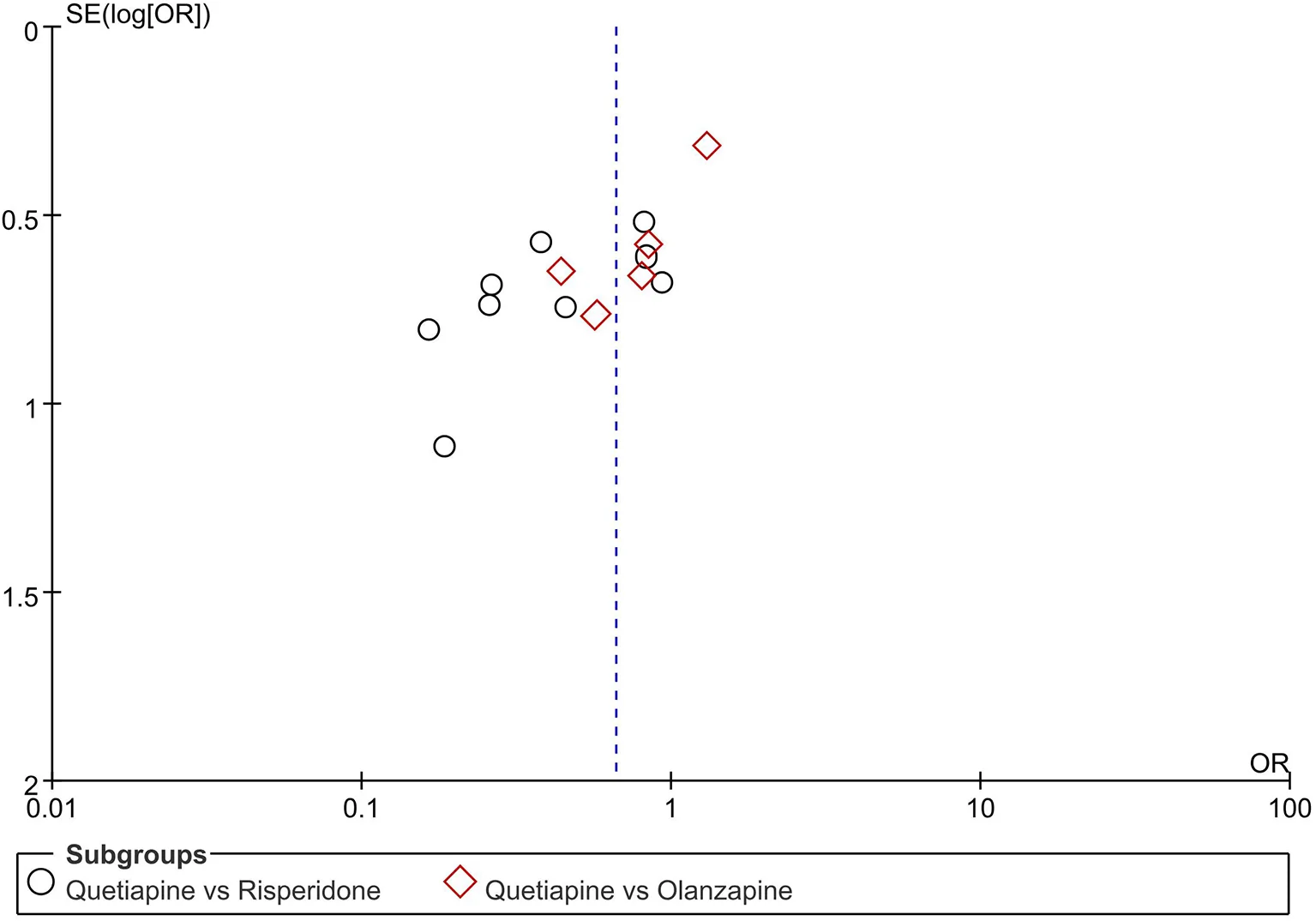
Risk of bias graph: summary of authors’ judgements across all included studies.

**Figure 3:**
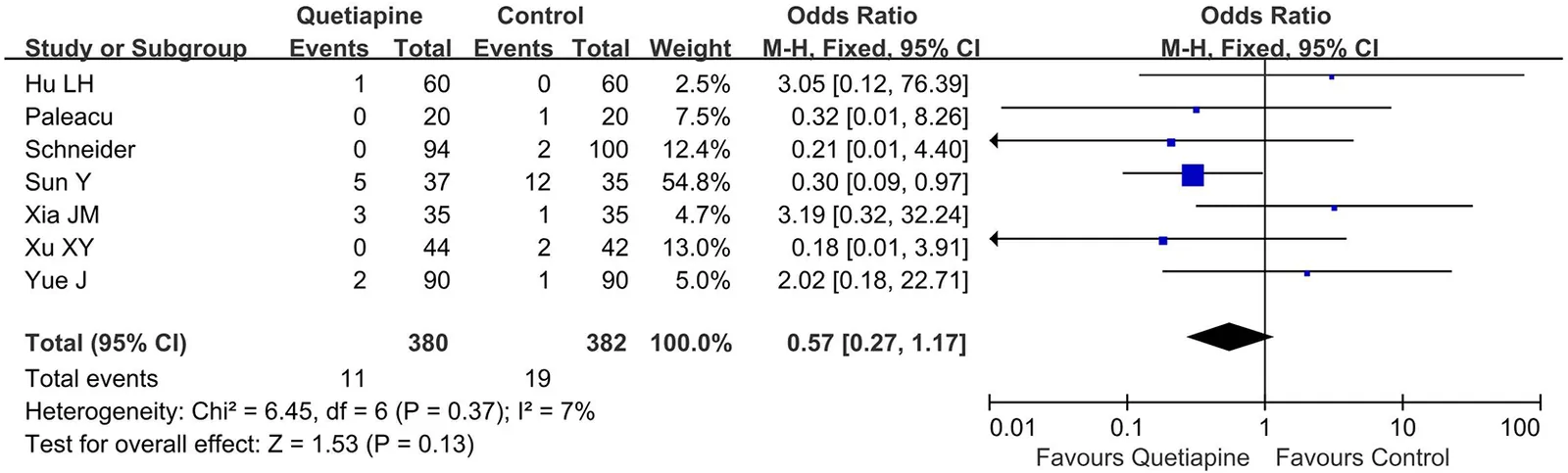
Risk of bias summary: authors’ judgements for each individual study.

### Primary efficacy outcome: behavioral and psychological symptoms

The comparative efficacy of quetiapine versus other atypical antipsychotics (risperidone and olanzapine) for the management of behavioral and psychological symptoms in Alzheimer’s disease was evaluated (**Figure 4**). In the efficacy analysis, the event of interest was defined as the absence of clinical improvement post-treatment. The meta-analysis result, therefore, represents the odds of treatment failure (i.e., no improvement) with quetiapine compared to other antipsychotics. The pooled analysis across 16 studies, involving a total of 792 patients in the quetiapine group and 786 patients in the control group, demonstrated a statistically significant overall treatment effect favoring quetiapine. The overall pooled Odds Ratio (OR) for treatment failure (i.e., no clinical improvement) was 0.66 (95% CI: 0.49 to 0.89, P = 0.006). This indicates that quetiapine was associated with significantly lower odds of treatment failure, representing a superior efficacy profile compared to other antipsychotics. In the pre-specified subgroup analysis, quetiapine showed a statistically significant superiority compared to risperidone, based on data from 10 studies (469 vs. 457 patients), with a pooled OR of 0.50 (95% CI: 0.33 to 0.75, P = 0.0009). In the comparison with olanzapine, which included 6 studies (323 vs. 329 patients), the point estimate also favored quetiapine (OR 0.90), but the 95% confidence interval (0.58 to 1.38, P = 0.62) crossed 1, indicating that the difference did not reach conventional statistical significance. The test for subgroup differences suggested significant difference in the treatment effect between the risperidone and olanzapine comparisons (P = 0.05, I^2^ = 73.7). Random-effects model was therefore adopted for the pooled analysis.

**Figure 4:**
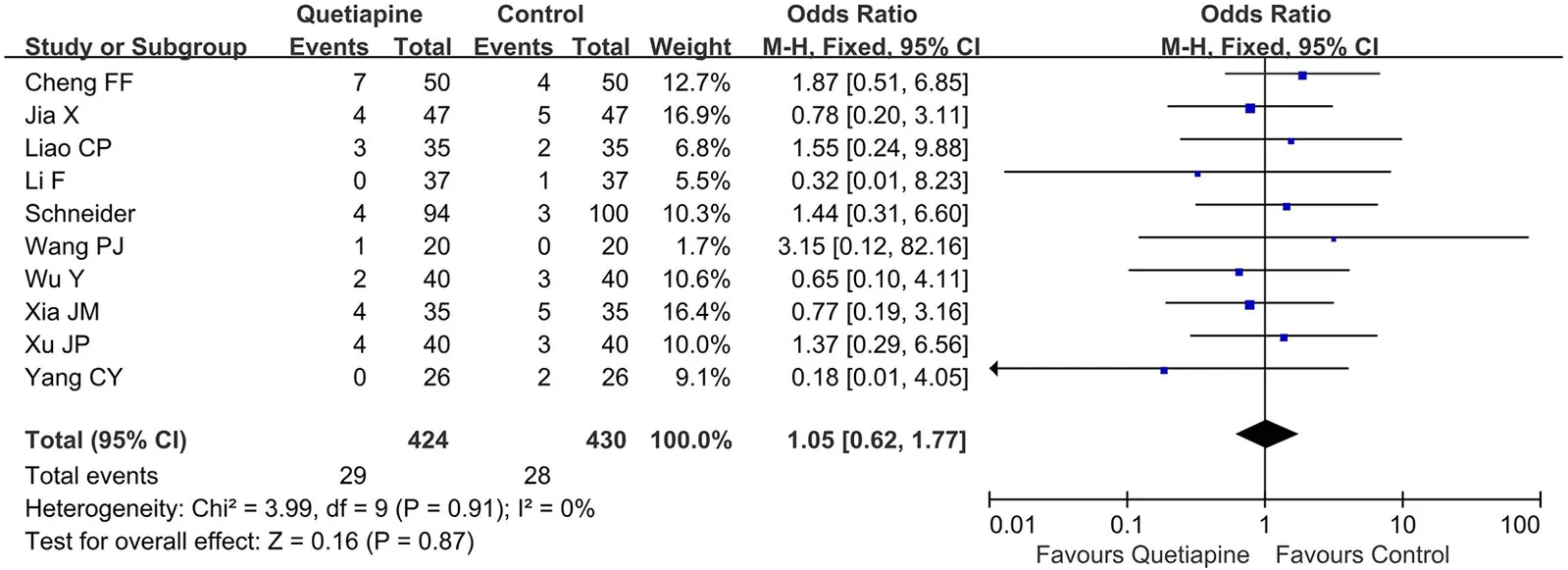
Forest plot for the comparative efficacy of quetiapine versus other antipsychotics (risperidone and olanzapine).

### Safety and tolerability outcomes

The safety profile of quetiapine was assessed through a meta-analysis of six specific adverse events. The analysis of somnolence (**Figure 5**) across 10 studies (515 quetiapine, 519 control patients) found no statistically significant difference in risk between groups, with a pooled OR of 0.90 (95% CI: 0.60 to 1.36, P = 0.62) and no heterogeneity (I² = 0%). For dizziness (**Figure 6**), data from 12 studies (547 quetiapine, 547 control) also showed no significant difference, yielding a pooled OR of 0.86 (95% CI: 0.51 to 1.43, P = 0.55) with no heterogeneity (I² = 0%). The analysis of constipation (**Figure 7**), involving 10 studies (424 quetiapine, 430 control patients), resulted in a pooled OR of 1.05 (95% CI: 0.62 to 1.77, P = 0.87) and no heterogeneity. In contrast, the analysis of extrapyramidal symptoms (EPS) (**Figure 8**) across 8 studies (330 quetiapine, 336 control patients) demonstrated a statistically significant and substantial reduction in risk for quetiapine, with a pooled OR of 0.17 (95% CI: 0.09 to 0.33, P < 0.00001) and no heterogeneity (I² = 0%). For nausea (**Figure 9**), data from 7 studies (311 quetiapine, 307 control) showed a non-significant trend favoring quetiapine, with a pooled OR of 0.56 (95% CI: 0.27 to 1.20, P = 0.14) and no heterogeneity. Finally, the analysis of tremor (**Figure 10**) in 7 studies (380 quetiapine, 382 control) found no statistically significant difference between the treatment groups, with a pooled OR of 0.57 (95% CI: 0.27 to 1.17, P = 0.13) and low heterogeneity (I² = 7%).

**Figure 5:**
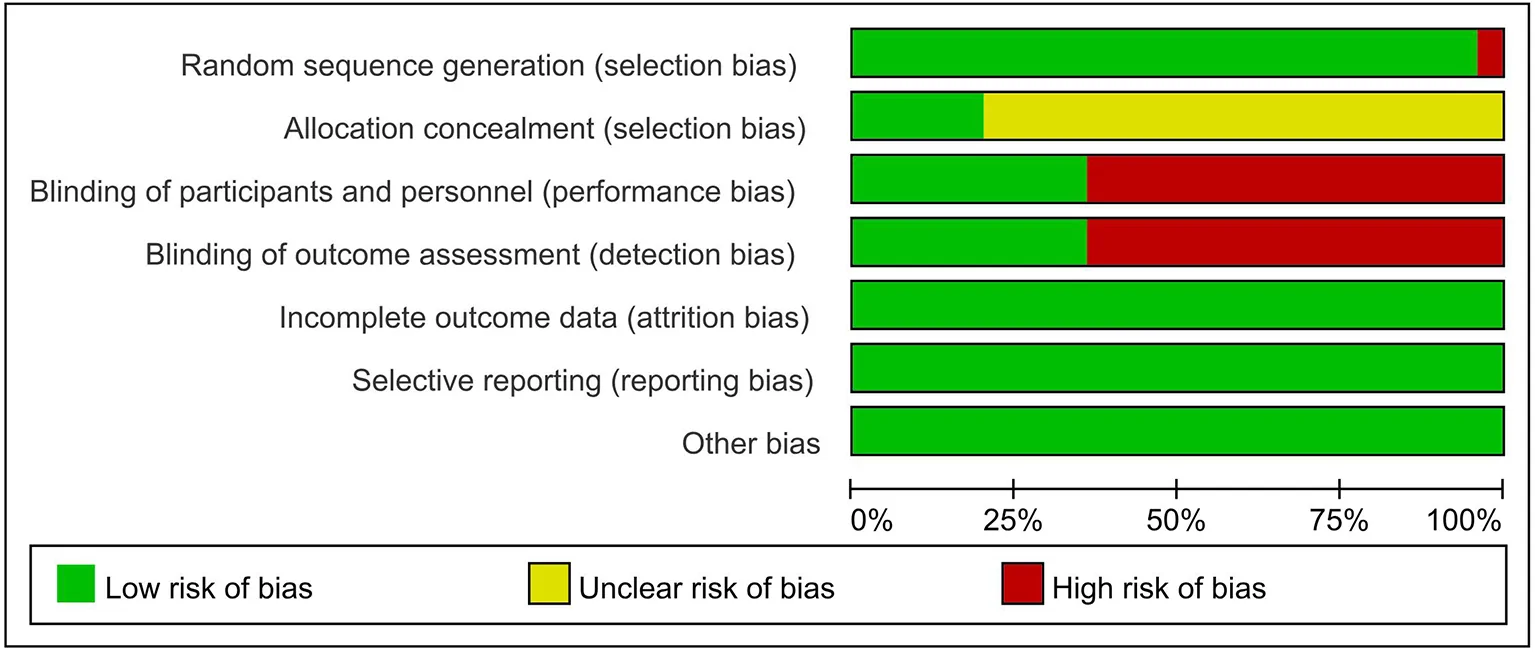
Forest plot for the risk of somnolence.

**Figure 6:**
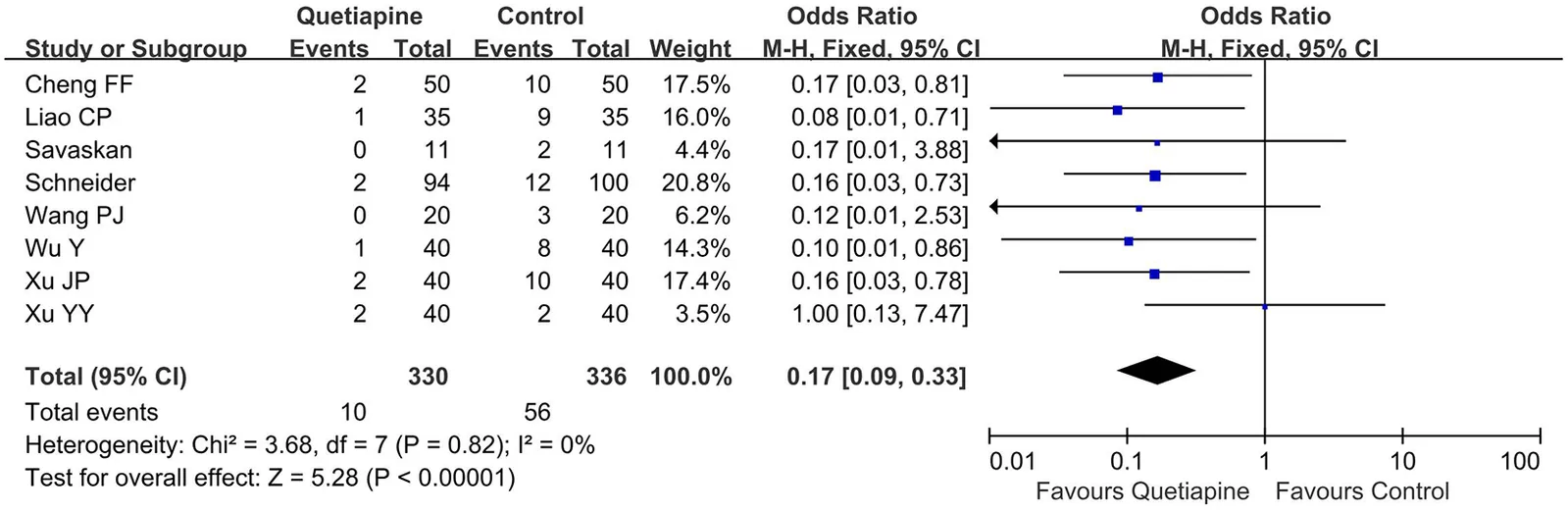
Forest plot for the risk of dizziness.

**Figure 7:**
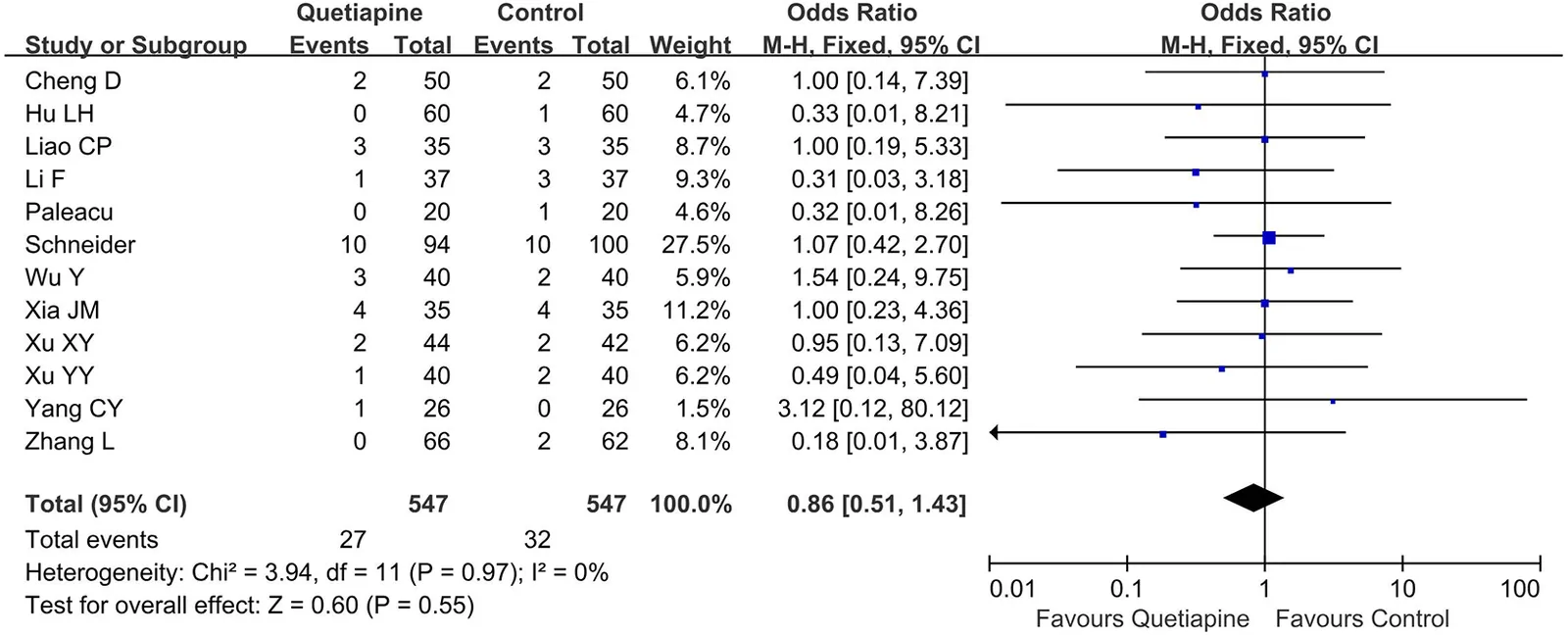
Forest plot for the risk of constipation.

**Figure 8:**
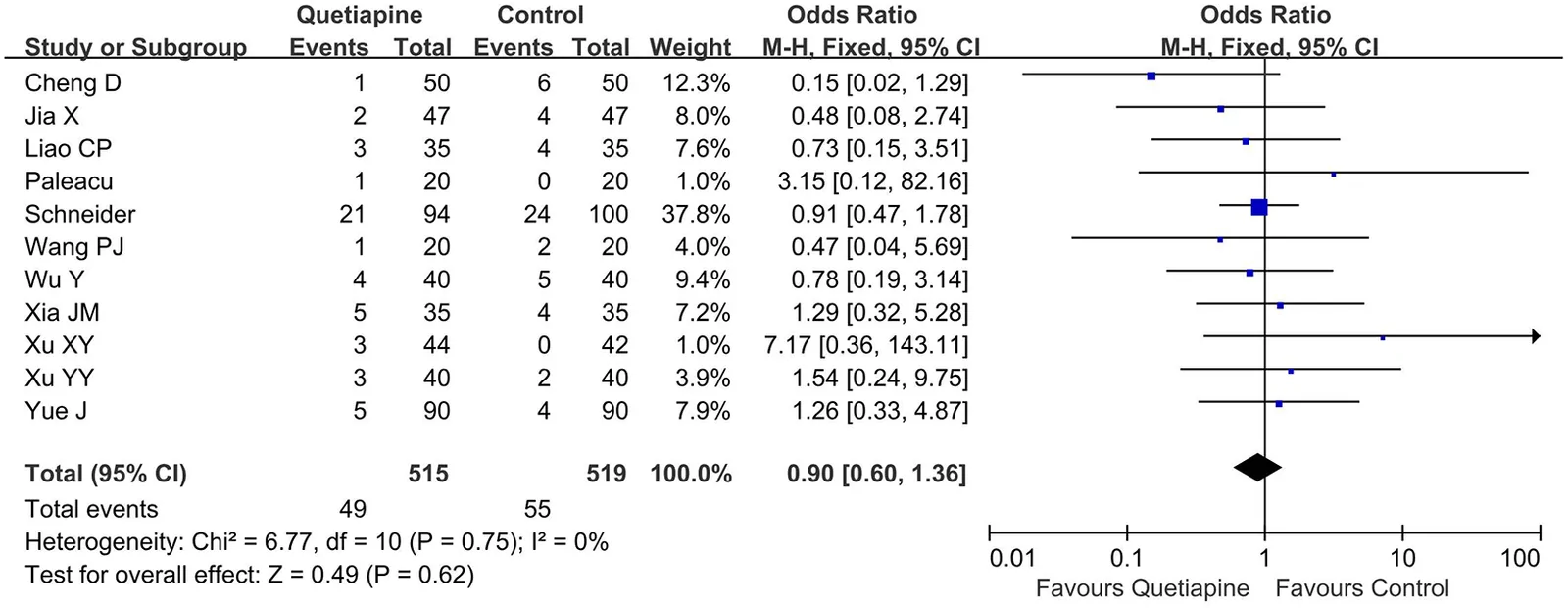
Forest plot for the risk of extrapyramidal symptoms (EPS).

**Figure 9:**
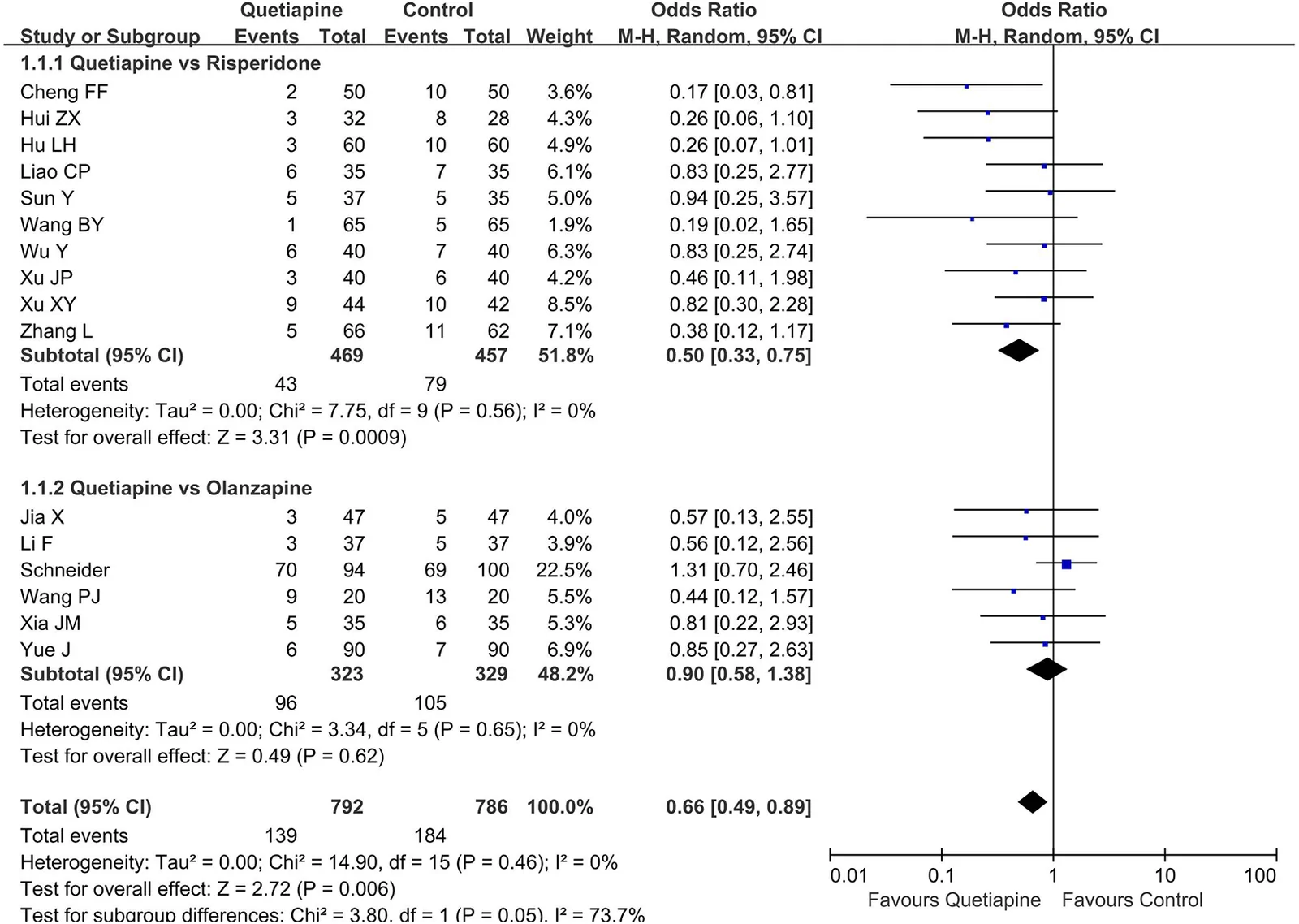
Forest plot for the risk of nausea.

**Figure 10:**
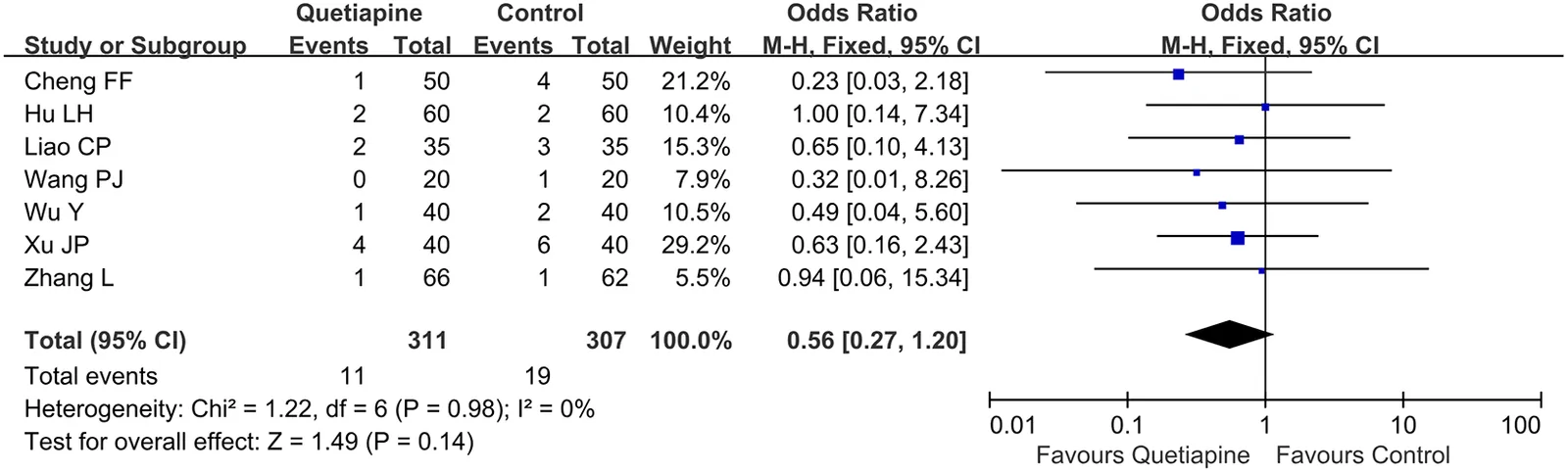
Forest plot for the risk of tremor.

### Publication bias

Potential publication bias for the primary efficacy outcome was evaluated using a funnel plot (**Figure 11**). Visual inspection of the funnel plot showed a symmetrical distribution, indicating a low likelihood of substantial publication bias.

**Figure 11:**
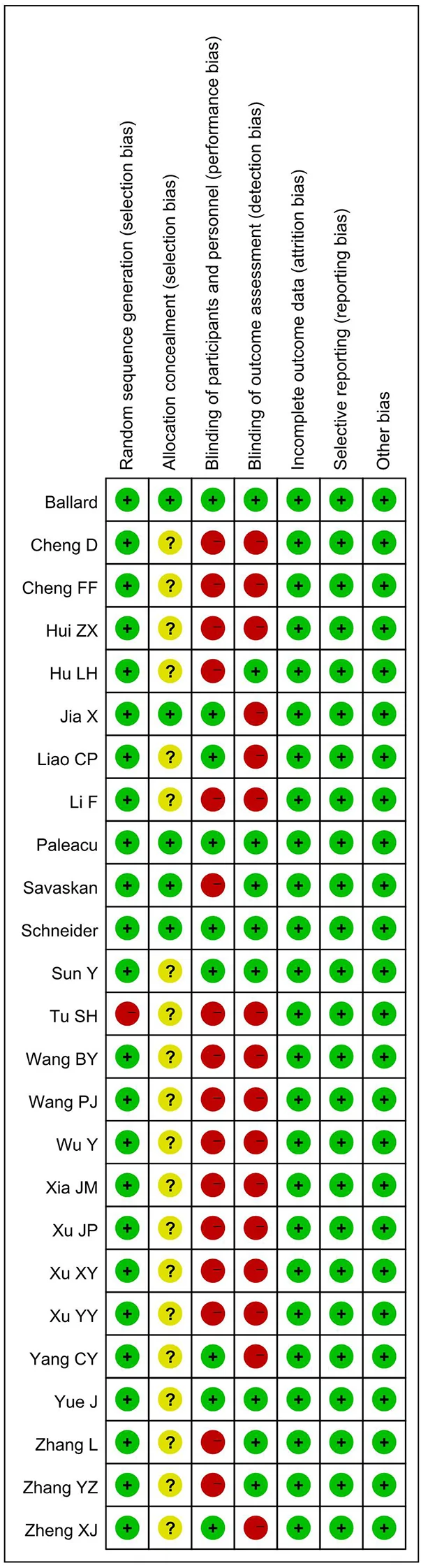
Funnel plot to assess potential publication bias for the primary efficacy outcome.

## Discussion

This systematic review and meta-analysis directly compared the efficacy and safety of quetiapine versus other active pharmacological agents, primarily risperidone and olanzapine, for managing BPSD in patients specifically diagnosed with AD. The principal findings indicate that, within the context of the included RCTs, quetiapine demonstrated statistically significant superior efficacy, defined as a lower odd of treatment failure, compared to the pooled group of other atypical antipsychotics. This superior efficacy was particularly evident and significant in the head-to-head comparison with risperidone. Regarding safety, quetiapine exhibited a comparable profile to other agents for most common adverse events, such as somnolence, dizziness, and constipation. Crucially, however, it was associated with a markedly and significantly lower risk of extrapyramidal symptoms (EPS), a finding of paramount importance for treating the vulnerable elderly AD population. These results provide a nuanced, comparative evidence synthesis that can help clinicians navigate the challenging risk-benefit assessment when pharmacotherapy for severe BPSD becomes necessary.

At first glance, our findings regarding the superior efficacy of quetiapine appear to contrast with the results of the large-scale Clinical Antipsychotic Trials of Intervention Effectiveness-Alzheimer’s Disease (CATIE-AD) study, which reported no significant difference in clinical global improvement between quetiapine, olanzapine, risperidone, and placebo in outpatients with AD (40). However, several key methodological differences may explain this discrepancy. First, the CATIE-AD trial primarily enrolled community-dwelling outpatients with relatively moderate symptom severity, whereas the RCTs included in our meta-analysis may have encompassed a higher proportion of acutely agitated or hospitalized patients, in whom antipsychotic effects on behavioral stabilization are more pronounced. Second, the mean dose of quetiapine in CATIE-AD was notably low (56.5 mg/day), which may have been potentially subtherapeutic to achieve optimal efficacy in many patients, whereas effective dosing varied across the trials in our analysis. Furthermore, the primary outcome in CATIE-AD was time to treatment discontinuation for any reason, a composite endpoint heavily influenced by early adverse events and caregiver burden, whereas our efficacy assessment focused strictly on the achievement of a 30% reduction in BEHAVE-AD scores, providing a more sensitive measure of direct symptomatic control.

Our primary efficacy analysis, pooling data from 16 studies, yielded an odds ratio (OR) of 0.66 for treatment failure, favoring quetiapine. This suggests that patients receiving quetiapine were about 66% as likely to experience a lack of clinical improvement compared to those receiving other antipsychotics. The absence of statistical heterogeneity (I²=0%) across these diverse studies strengthens the robustness of this pooled estimate. The pre-specified subgroup analyses offer further granularity. The significant superiority of quetiapine over risperidone (OR 0.50) is a notable finding, as risperidone is often considered a first-line atypical antipsychotic for BPSD due to its established efficacy data (12). The point estimate for the comparison with olanzapine also favored quetiapine (OR 0.90), though the confidence interval crossed unity. The non-significant test for subgroup differences suggests that the treatment effect is likely consistent across these comparators, with the wider confidence interval for the olanzapine comparison possibly attributable to the smaller number of studies and patients in this subgroup.

These findings contribute to resolving the ambiguity in the existing literature regarding quetiapine’s efficacy. While some earlier individual RCTs and narrative reviews questioned quetiapine’s potency, particularly at lower doses (14), our quantitative synthesis of direct comparative trials suggests that, within the dosing ranges studied, it is at least as efficacious, and potentially more so, than its main alternatives (41). This may be partially explained by its distinct receptor profile. Quetiapine’s lower D2 receptor occupancy and higher affinity for serotonergic (5-HT2A) and adrenergic (α1) receptors might translate to a broader spectrum of activity against the multifaceted symptom cluster of BPSD, which includes not only psychosis but also agitation, aggression, and anxiety (8). Furthermore, its low propensity for EPS (discussed below) may improve tolerability and adherence, indirectly contributing to better observed efficacy outcomes in clinical trials (42).

Our results both align with and contrast certain aspects of previous meta-analyses. Earlier seminal meta-analyses primarily established the class-wide efficacy of atypical antipsychotics over placebo and highlighted their associated risks, including mortality (9, 10). More recent network meta-analyses (NMAs) have attempted to rank these agents. For instance, a 2019 NMA by Yunusa et al. found risperidone and olanzapine to be among the most effective agents, while quetiapine’s efficacy profile was less distinct, though it was noted for a relatively favorable safety profile (11). A key distinction is that many such analyses combine all dementia types, potentially diluting or confounding signals specific to AD pathophysiology. Our analysis, by strictly focusing on AD, provides a more homogenous and clinically relevant comparison.

The finding of quetiapine’s potentially superior efficacy relative to risperidone requires careful contextualization. Some studies suggest risperidone may have a more pronounced effect on specific, severe symptoms like aggression (12). Our outcome was a global measure of treatment response (e.g., ≥30% reduction on BEHAVE-AD), which captures overall improvement. It is plausible that while risperidone might be superior for a specific target symptom, quetiapine’s broader pharmacological action leads to a more comprehensive global response in the heterogeneous AD-BPSD population. This hypothesis warrants investigation in future studies with detailed symptom-domain analyses. Another contributing factor to the observed difference could be the higher incidence of EPS with risperidone, which might lead to dose reduction, discontinuation, or use of anticholinergic medications (which can worsen cognition), thereby attenuating its perceived effectiveness in a “real-world” trial setting (43–45).

The safety analysis reveals the most compelling argument for considering quetiapine’s distinct position, associated with an 83% relative reduction in the odds of EPS. EPS are a major deterrent to antipsychotic use in the elderly, causing significant discomfort, increasing fall risk, and potentially exacerbating functional decline. This finding is consistent with quetiapine’s well-known pharmacodynamic profile of transient, low D2 receptor occupancy, which underlies its low EPS liability even at higher doses (46). In contrast, risperidone exhibits dose-dependent D2 occupancy closely linked to EPS risk, and olanzapine has a moderate risk, often mediated through its potent muscarinic antagonism.

For other common adverse events, somnolence, dizziness, constipation, nausea, and tremor, no statistically significant differences were found. This is an important finding, as it counters the notion that quetiapine’s favorable EPS profile comes at the expense of significantly greater sedation or metabolic disturbance. The trend towards lower nausea with quetiapine, though not significant, is also physiologically plausible given its potent 5-HT2A antagonism, which can have anti-emetic properties. The comparable rates of somnolence, a known effect of quetiapine’s H1 receptor blockade, suggest that the sedation profiles of these agents, while mediated by different receptor mechanisms, are not drastically different in practice within the studied dosing regimens for BPSD. It must be emphasized that our analysis was limited to short- to medium-term trials; risks such as weight gain, metabolic syndrome, and tardive dyskinesia, which are critical for long-term management, could not be adequately assessed (47).

The integrated efficacy and safety profile observed here supports a reconsideration of the treatment algorithm for severe AD-related BPSD. When non-pharmacological interventions fail and an antipsychotic is deemed necessary, the choice must balance the need for symptom control with the imperative to minimize harm, particularly neurologic harm. Our data suggest that quetiapine offers a favorable balance, providing notable efficacy signal with a significantly reduced risk of EPS. This makes it a particularly viable option for patients with Parkinsonian features, those at high fall risk, or individuals who have previously experienced EPS with other antipsychotics. The comparable efficacy to olanzapine, coupled with a likely better metabolic profile (though not directly assessed here), and the significant efficacy advantage over risperidone in our analysis, position quetiapine as a carefully considered atypical antipsychotic choice in this specific population.

These findings should be implemented within the overarching framework of cautious antipsychotic use. They do not advocate for widespread or first-line pharmacological treatment but rather inform the best choice when such treatment is unavoidable. Therapy should always be initiated at the lowest effective dose, regularly re-evaluated for continued necessity, and accompanied by ongoing non-pharmacological support (6, 7).

When interpreting these results, several limitations must be acknowledged. Despite the sample size achieved by including 25 RCTs, the methodological quality and risk of bias varied among the constituent studies. Furthermore, the diagnosis of AD and the assessment of BPSD were not uniform across all trials, and the potential for unblinding due to the distinct side-effect profiles of the antipsychotics cannot be entirely excluded. Additionally, while our analysis provides a contemporary comparison of these agents, the long-term safety of quetiapine and other atypical antipsychotics, particularly concerning metabolic adverse events, cerebrovascular risks, and mortality, was not comprehensively captured in this analysis. Clinicians must weigh the modest efficacy benefits observed in our study against the well-documented safety risks associated with this drug class in the elderly. In addition, a considerable proportion of included trials evaluated quetiapine in combination with cholinesterase inhibitors rather than as monotherapy. Consequently, the observed global efficacy may partly reflect synergistic effects, and our findings should not be interpreted as endorsing quetiapine monotherapy efficacy in isolation. Moreover, our harmonization of the CATIE-AD trial’s CGIC scores into binary responder data relied on published aggregate totals rather than individual participant data, which may introduce outcome ascertainment bias. Future meta-analyses with individual participant data (IPD) are warranted to validate these aggregated extrapolations. Ultimately, our findings suggest that while quetiapine may offer a favorable neurological tolerability profile and superior short-term efficacy for severe BPSD compared to other atypicals, it should remain a second-line intervention, strictly reserved for cases where severe, persistent, or dangerous symptoms have proven refractory to exhaustive non-pharmacological approaches.

In conclusion, this meta-analysis of head-to-head RCTs provides evidence that in patients with Alzheimer’s disease suffering from behavioral and psychological symptoms, quetiapine is associated with superior global efficacy compared to other atypical antipsychotics, particularly risperidone, while maintaining a comparable overall adverse event profile. Its most distinguishing and clinically advantageous feature is a substantially lower risk of extrapyramidal symptoms. These findings help clarify quetiapine’s role in the treatment algorithm for AD-related BPSD, suggesting it is an effective and neurologically safer alternative to other commonly used atypical antipsychotics. This information can assist clinicians in making more personalized, risk-aware decisions when pharmacological management becomes necessary. Future research should prioritize large, well-blinded, long-term comparative effectiveness trials that include comprehensive metabolic and functional outcomes (48).

## Data Availability

The original contributions presented in the study are included in the article/supplementary material. Further inquiries can be directed to the corresponding author.

## References

[1] Cerejeira J Lagarto L Mukaetova-Ladinska EB . Behavioral and psychological symptoms of dementia. Front Neurol. (2012) 3:73. doi: 10.3389/fneur.2012.00073 22586419 PMC3345875

[2] Zhao QF Tan L Wang HF Jiang T Tan MS Tan L . The prevalence of neuropsychiatric symptoms in Alzheimer's disease: Systematic review and meta-analysis. J Affect Disord. (2016) 190:264–71. doi: 10.1016/j.jad.2015.09.069 26540080

[3] Okura T Plassman BL Steffens DC Llewellyn DJ Potter GG Langa KM . Neuropsychiatric symptoms and the risk of institutionalization and death: the aging, demographics, and memory study. J Am Geriatr Soc. (2011) 59:473–81. doi: 10.1111/j.1532-5415.2011.03314.x 21391937 PMC3088883

[4] Kales HC Gitlin LN Lyketsos CG . Assessment and management of behavioral and psychological symptoms of dementia. BMJ. (2015) 350:h369. doi: 10.1136/bmj.h369 25731881 PMC4707529

[5] Singh AS Naqvi AR Chanu MT . Microglial, astrocytic, oligodendrocyte, B-/T-cell and neutrophil dysregulation in neuroinflammation of Alzheimer's disease and related dementias. J Psychiatr Res. (2026) 195:225–43. doi: 10.1016/j.jpsychires.2026.01.050 41637998

[6] Kales HC Lyketsos CG Miller EM Ballard C . Management of behavioral and psychological symptoms in people with Alzheimer's disease: an international Delphi consensus. Int Psychogeriatr. (2019) 31:83–90. doi: 10.1017/s1041610218000534 30068400

[7] Reus VI Fochtmann LJ Eyler AE Hilty DM Horvitz-Lennon M Jibson MD . The American psychiatric association practice guideline on the use of antipsychotics to treat agitation or psychosis in patients with dementia. Focus (Am Psychiatr Publ). (2017) 15:81–4. doi: 10.1176/appi.focus.15107 31997967 PMC6519629

[8] Wang J Yu JT Wang HF Meng XF Wang C Tan CC . Pharmacological treatment of neuropsychiatric symptoms in Alzheimer's disease: a systematic review and meta-analysis. J Neurol Neurosurg Psychiatry. (2015) 86:101–9. doi: 10.1136/jnnp-2014-308112 24876182

[9] Schneider LS Dagerman KS Insel P . Risk of death with atypical antipsychotic drug treatment for dementia: meta-analysis of randomized placebo-controlled trials. JAMA. (2005) 294:1934–43. doi: 10.1001/jama.294.15.1934 16234500

[10] Ma H Huang Y Cong Z Wang Y Jiang W Gao S . The efficacy and safety of atypical antipsychotics for the treatment of dementia: a meta-analysis of randomized placebo-controlled trials. J Alzheimers Dis. (2014) 42:915–37. doi: 10.3233/jad-140579 25024323

[11] Yunusa I Alsumali A Garba AE Regestein QR Eguale T . Assessment of reported comparative effectiveness and safety of atypical antipsychotics in the treatment of behavioral and psychological symptoms of dementia: A network meta-analysis. JAMA Netw Open. (2019) 2:e190828. doi: 10.1001/jamanetworkopen.2019.0828 30901041 PMC6583313

[12] Ballard C Waite J . The effectiveness of atypical antipsychotics for the treatment of aggression and psychosis in Alzheimer's disease. Cochrane Database Syst Rev. (2006) 2006:CD003476. doi: 10.1002/14651858.cd003476.pub2 16437455 PMC11365591

[13] Sultana J Chang CK Hayes RD Broadbent M Stewart R Corbett A . Associations between risk of mortality and atypical antipsychotic use in vascular dementia: a clinical cohort study. Int J Geriatr Psychiatry. (2014) 29:1249–54. doi: 10.1002/gps.4101 24633896

[14] Tariot PN Schneider L Katz IR Mintzer JE Street J Copenhaver M . Quetiapine treatment of psychosis associated with dementia: a double-blind, randomized, placebo-controlled clinical trial. Am J Geriatr Psychiatry. (2006) 14:767–76. doi: 10.1097/01.jgp.0000196628.12010.35 16905684

[15] Terao I Kodama W . Comparative efficacy and tolerability of multiple antipsychotics across varying doses for neuropsychiatric symptoms of dementia including alzheimer's disease: A dose-response model-based network meta-analysis. Acta Psychiatr Scand. (2026) 153:82–94. doi: 10.1111/acps.70051 41276485

[16] Yue J . Clinical efficacy and side effects of olanzapine and quetiapine in the treatment of behavioral and psychological symptoms of Alzheimer's disease. Chin J Mod Drug Appl. (2022) 16:128–9.

[17] Cheng FF Chen W Ma Z . Analysis of the effect of quetiapine in the treatment of mental and behavioral symptoms in patients with Alzheimer's disease. Psychol Monthly. (2022) 17:40–1.

[18] Zhang YZ Zhang JY Fang HL Ma ZZ Wang SM Sun J . Comparative study for effects of olanzapine and quetiapine on blood homocysteine in patients with BPSD of Alzheimer's dementia. China J Health Psychol. (2020) 28:325–8.

[19] Wang BY Chen Q Sun LB Zeng JM Chen HF Feng JF . A clinical controlled study of quetiapine and risperidone in the treatment of mental behavior symptoms of alzheimer's disease. Syst Med. (2020) 5:144–6.

[20] Zheng XJ . The analysis of therapeutic effects of psychological and behavioral symptoms for Alzheimer's Disease patients in neurology outpatient department. World Latest Medicne Inf. (2019) 19:112–3.

[21] Yang CY . Clinical value of olanzapine and quetiapine combined with donepezil in the treatment of behavioral and psychological symptoms of Alzheimer's disease. Chin J Mod Drug Appl. (2019) 13:185–7.

[22] Xu YY . Effect and safety of olanzapine or quetiapine combined with donepezil in the treatment of Alzheimer's disease. Med J Chin People's Health. (2019) 31:107–8.

[23] Wang PJ Liu C Xu HB Xu J . Study on quetiapine and olanzapine in the treatment of mental and behavioral symptoms associated with Alzheimer's disease. Med J Commun. (2019) 33:524–5.

[24] Jia X . Clinical comparison of olanzapine or quetiapine combined with donepezil in the treatment of psychotic symptoms of Alzheimer's disease. China Continuing Med Educ. (2019) 11:117–8.

[25] Chen D Chao ZW He YH Zhao YZ . Effect of donepezil combined with quetiapine on cognitive function and psychiatric symptoms in patients with Alzheimer's disease. J Int Psychiatry. (2019) 46:477–80.

[26] Tu SH Qu QS Mei G Li JJ Li HL . Efficacy and ECG analysis of olanzapine and quetiapine in the treatment of mental and behavioral symptoms of Alzheimer's disease. J Int Psychiatry. (2018) 45:1064–7.

[27] Li F Fan YB Wu CH Yang X Ren JG . Clinical efficacy and adverse reactions of quetiapine in the treatment of mental and behavioral symptoms of senile dementia. China Prac Med. (2017) 12:137–9.

[28] Hu LH . Clinical efficacy and adverse reactions of quetiapine in the treatment of senile dementia. China Continuing Med Educ. (2017) 9:179–80.

[29] Zhang R . Clinical observation of quetiapine in the treatment of mental and behavioral symptoms in senile dementia patients. J Qiqihar Univ Med. (2015) 36:2404–5.

[30] Xu JP Zhao XF Zhao ZR Zhang RJ . The analysis of therapeutic effects of psychological and behavioral symptoms for Alzheimer's Disease patients in neurology outpatient department. J Int Psychiatry. (2015) 42:48–50.

[31] Hui ZX . Application study on quetiapine in treatment of mental and behavioral symptoms of senile dementia. China Health Standard Manage. (2015) 6:113–4.

[32] Xu XY Ceng YY Zhang Y . Analyzing effect and safety of quetiapine and risperidone on mental and behavioral of alzheimer disease. J Clin Med Pract. (2013) 17:51–2.

[33] Liao CP Li XW Wang RY Wu DH Chen ZW . Analysis of clinical efficacy and safety of quetiapine and risperidone in the treatment of Alzheimer's disease patients with mental and behavioural disorders. Chin J Pract Nervous Dis. (2013) 16:11–2.

[34] Sun Y Liao QL Chen R Hu YJ . Queliapine treatment of behavioral and psychological symptoms of Alzheimer's disease clinical control study. World Health Digest. (2012) 9:96–7.

[35] Xia JM Li T Tang JL . Comparison of quetiapine and olanzapine in the treatment of mental and behavioral symptoms of Alzheimer's disease. Herald Med. (2011) 30:1293–4.

[36] Wu Y Qian FQ Yao HZ . A comparative study of quetiapine and risperidone for behavioral and psychological symptoms of Alzheimer's disease. J Clin Psychiatry. (2010) 20:274–5.

[37] Paleacu D Barak Y Mirecky I Mazeh D . Quetiapine treatment for behavioural and psychological symptoms of dementia in Alzheimer's disease patients: a 6-week, double-blind, placebo-controlled study. Int J Geriatr Psychiatry. (2008) 23:393–400. 10.1002/gps.1892 17879256

[38] Savaskan E Schnitzler C Schroder C Cajochen C Muller-Spahn F Wirz-Justice A . Treatment of behavioural, cognitive and circadian rest-activity cycle disturbances in Alzheimer's disease: haloperidol vs. quetiapine. Int J Neuropsychopharmacol. (2006) 9:507–16. doi: 10.1017/S1461145705006036 16316485

[39] Ballard C Margallo-Lana M Juszczak E Douglas S Swann A Thomas A . Quetiapine and rivastigmine and cognitive decline in Alzheimer's disease: randomised double blind placebo controlled trial. BMJ. (2005) 330:874. doi: 10.1136/bmj.38369.459988.8f 15722369 PMC556156

[40] Schneider LS Tariot PN Dagerman KS Davis SM Hsiao JK Ismail MS . Effectiveness of atypical antipsychotic drugs in patients with Alzheimer's disease. N Engl J Med. (2006) 355(15):1525–38. doi: 10.1056/NEJMoa061240 17035647

[41] Tampi RR Tampi DJ Balachandran S Srinivasan S . Antipsychotic use in dementia: a systematic review of benefits and risks from meta-analyses. Ther Adv Chronic Dis. (2016) 7:229–45. doi: 10.1177/2040622316658463 27583123 PMC4994396

[42] MT. SAaC . Epigenetic regulation of neuroinflammation in dementia of alzheimer's disease and other forms of dementias. EC Clin Med Case Rep. (2025) 8:01–14. doi: 10.31080/ECCMC.2025.08.01047

[43] Qasim HS Simpson MD . A narrative review of studies comparing efficacy and safety of citalopram with atypical antipsychotics for agitation in behavioral and psychological symptoms of dementia (BPSD). Pharm (Basel). (2022) 10(3):61. doi: 10.3390/pharmacy10030061 35736776 PMC9228736

[44] Carson S McDonagh MS Peterson K . A systematic review of the efficacy and safety of atypical antipsychotics in patients with psychological and behavioral symptoms of dementia. J Am Geriatr Soc. (2006) 54:354–61. doi: 10.1111/j.1532-5415.2005.00566.x 16460391

[45] Daiello LA Beier MT Hoffmann VP Kennedy JS . Pharmacotherapy of behavioral and psychological symptoms of dementia: a review of atypical antipsychotics. Consult Pharm. (2003) 18:138–52. 16563055

[46] Nemeroff CB Kinkead B Goldstein J . Quetiapine: preclinical studies, pharmacokinetics, drug interactions, and dosing. J Clin Psychiatry. (2002) 63:5–11. 12562141

[47] Maust DT Kim HM Seyfried LS Chiang C Kavanagh J Schneider LS . Antipsychotics, other psychotropics, and the risk of death in patients with dementia: number needed to harm. JAMA Psychiatry. (2015) 72:438–45. doi: 10.1001/jamapsychiatry.2014.3018 25786075 PMC4439579

[48] Singh AS NA Chanu MT . Neuroinflammation and progress in clinical trials for the treatment of Alzheimer's disease and related dementias: An update. Innovative Medicines Omics. (2025) 2:36–50. doi: 10.36922/imo025050007

